# Dual spin nodal box structure in ternary ferromagnet K_3_NiCl_6_ with broad topological surface states†

**DOI:** 10.1039/d4ra06808d

**Published:** 2024-11-15

**Authors:** Yang Li

**Affiliations:** a Aviation and Automobile School, Chongqing Youth Vocational & Technical College Chongqing China liyang@cqwu.edu.cn; b College of Physics, Chongqing University Chongqing China

## Abstract

In recent years, there has been a discernible shift in research focus towards investigating the intricate interplay between topological states and intrinsic magnetic orders within the realm of condensed matter physics. Embedded within this evolving landscape, our study unveils an intriguing spin nodal box structure within the ferromagnetic compound K_3_NiCl_6_, manifesting under both spin directions. This distinctive configuration features a simplistic two-band crossing pattern that stands distinctly apart from other bands, making it amenable to both experimental validation and thorough theoretical exploration. The elucidation of the formation mechanism behind this spin nodal box has been meticulously achieved through systematic symmetry analyses, while the criteria for band crossings have been rigorously scrutinized using the Hubbard *U* method. Broad distribution of the surface state is derived from the Wannier tight-binding Hamiltonian and it is also well separated from the bulk band projection. More importantly, the band structure and the correlated surface states can be properly maintained even under the spin orbital coupling effect, attributed to the contributions from the light element orbitals associated with the relevant topological bands. Overall, the K_3_NiCl_6_ compound demonstrates a diverse array of advantages, positioning it as a promising candidate for experimental investigation, particularly in relation to its magnetic properties.

## Abstract 2

In recent years, there has been a discernible shift in research focus towards investigating the intricate interplay between topological states and intrinsic magnetic orders within the realm of condensed matter physics.

## Graphical abstract

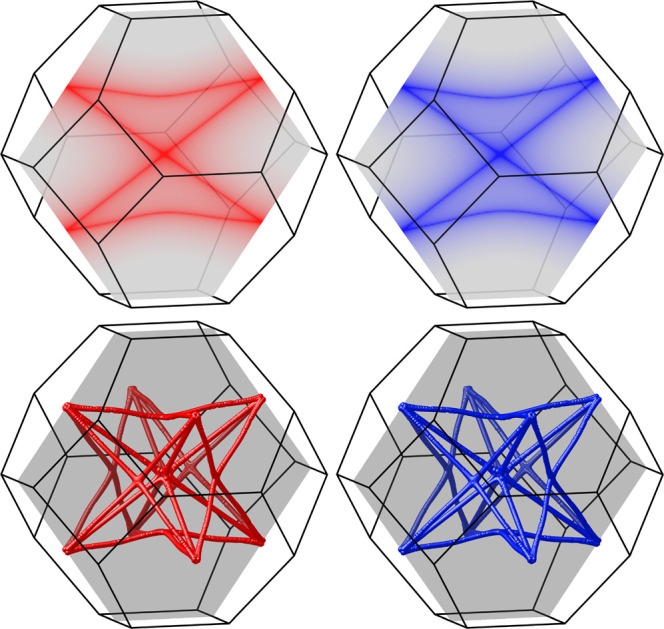

## Introduction

Significant advancements have been achieved in the realm of condensed matter science and solid-state physics since the initial unveiling of topological insulators. ^1–4^ Central to this progress has been the emergence and refinement of topological band theory. ^5–12^ This transformative framework elucidates the intricate interplay between topological states present in crystalline materials, their associated symmetry operations, and the constraints imposed by band topology. This theoretical topological band theory has served as a cornerstone in the exploration of the topological characteristics exhibited by diverse material systems, ^13,14^ spanning both experimental investigations and theoretical analyses. The trajectory of research in this field has transitioned from a primary focus on topological insulators to encompass semi-metals and even half-metals. ^15–27^ Unlike conventional topological insulators, topological features in semi-metals or half-metals are defined by linear band crossings. ^28–31^ A single point crossing denotes a nodal point, ^32–36^ while line and surface crossings correspond to nodal lines ^37–42^ and nodal surfaces, ^43–48^ respectively. This taxonomy encapsulates the diverse manifestations of topological phenomena within materials, offering a comprehensive framework for characterizing and studying their unique properties and behaviors. When these crossings occur near the Fermi energy level, they yield low-energy excitations that serve as analogs to particles in high-energy physics, offering a simplified means to explore fundamental physics within condensed matter. ^49–52^ This field continues to attract significant attention and research efforts.

Topological characteristics manifested in semi-metals and half-metals showcase distinctive surface states reminiscent of the surface Dirac cone state observed in topological insulators. ^53–55^ The surface states exhibited by different elements present a spectrum of conditions: Fermi arc states that link isolated nodal points and drumhead surface states that encircle nodal lines. Conceptually, nodal lines can be visualized as continuous sequences of nodal points, while drumhead surfaces serve as amalgamations of multiple Fermi arcs. Notably, nodal surfaces lack clearly defined surface states, setting them apart from their counterparts. Recent research endeavors have honed in on half-metals due to their capacity for manipulating topological properties *via* magnetic interactions and configurations. The convergence of topology and magnetism has opened up novel avenues for exploration, promising to reshape the landscape of both disciplines. Within the realm of topological states, nodal lines stand out for their remarkable flexibility and versatility, underpinning a host of distinct physical phenomena.^36–39^ These include drumhead surface states, non-dispersive Landau energy levels, specific long-range Coulomb interactions, and characteristic Friedel oscillations. Researchers are actively engaged in the quest for innovative topological nodal line phases within half-metal materials, driven by the quest to unveil new properties and harness their potential applications in magnetic systems. This pursuit not only expands our understanding of topological materials but also paves the way for groundbreaking advancements at the intersection of topology and magnetism.

To date, a wide array of nodal line states has been both proposed and discovered in various magnetic systems. Notably, nontrivial topological structures such as Hopf and Chain link semimetal states have been identified in ferromagnetic full Heusler compounds like Co_2_MnGa and Co_2_MnAl. This identification was achieved by engineering the mirror eigenvalues of the conduction and valence bands across two perpendicular mirror planes. ^56^ Furthermore, ideal Weyl semimetal characteristics have emerged with a ferromagnetic ground state in centrosymmetric tetragonal structures such as β-V_2_OPO_4_, Co_2_S_2_Tl, and Fe_2_S_2_X (X = Al, Ga, In). Remarkably, the type of Weyl node lines within these materials can be manipulated through the variation of magnetization direction, under the influence of spin–orbit coupling. ^57^ In another instance, spin-polarized Weyl chains have been discovered in the ferromagnetic compound Li_3_(FeO_3_)_2_, featuring one Weyl loop of type I and another of a hybrid type. ^58^ Moreover, complex topological nodal loop states have been reported in the local antiferromagnetic electride Ba_4_Al_5_ e^−^, where these topological fermions are intriguingly contributed by the excess electron localized within the crystal cavity. ^59^ Although significant progress has been made in exploring the complexities of topological nodal line states within half-metallic systems, the catalog of known materials remains limited and the theoretical grasp of these phenomena is not yet exhaustive.

A critical imperative exists to unearth additional materials characterized by not only simple band configurations, particular those with the most straightforward condition, but also resilient surface states. In light of the ongoing quest within this context, we advocate for the ternary ferromagnet K_3_NiCl_6_ as an exemplary contender, showcasing spin nodal line box structures in both spin channels. Our theoretical investigations and model analyses unveil that these nodal box configurations in K_3_NiCl_6_ involve merely two bands, epitomizing the most simplistic scenario of band crossings. Noteworthy surface states are discernible, distinctly isolated from the bulk band projection and extensively dispersed throughout the Brillouin zone. Of paramount significance is the preservation of these spin nodal boxes even in the presence of spin–orbit coupling, underscoring the robustness of these features. The distinctive attributes exhibited by K_3_NiCl_6_ position it as a propitious candidate for experimental scrutiny, presenting an auspicious platform for delving into the realm of exotic topological properties. The unique characteristics of this material offer exceptional prospects for exploration, promising fresh insights into the rich tapestry of topological phenomena.

## Computational methodology

The study employed first-principles calculations conducted utilizing the Vienna *Ab initio* Simulation Package (VASP) ^60–62^ within the framework of density functional theory (DFT). ^63–65^ The Perdew–Burke–Ernzerhof (PBE) functional, operating under the generalized gradient approximation (GGA), ^66^ served as the foundational tool for this investigation. To strike a balance between computational efficiency and precision, the projector augmented wave (PAW) method was harnessed, ^67,68^ with a plane-wave basis energy cutoff established at 520 eV. Sampling of the Brillouin zone was achieved through a 12 × 12 × 12 Monkhorst–Pack *k*-point mesh. Rigorous self-consistent convergence criteria were enforced, setting energy convergence at 1 × 10^−6^ eV and force convergence at 1 × 10^−3^ eV Å^−1^. In order to effectively capture correlation effects within transition metal elements, particularly the behavior of Ni, the DFT + *U* method was called upon. ^69–71^ It is noteworthy that adjustments to the effective *U* value, spanning a range from 1 to 3 eV, did not sway the core findings of this investigation. The extraction and analysis of electronic band structures were meticulously carried out using the VASPKIT high-throughput analysis package. ^72^ Surface states were computed through the utilization of maximally localized Wannier functions, structured using the WANNIER90 code and further processed with the WANNIERTOOLS package. ^73–75^ This comprehensive computational approach facilitated a thorough examination of the material system under investigation, ensuring a robust foundation for the study's conclusions.

## Results and discussions

### Crystal structure and magnetic configuration

**Fig. 1:**
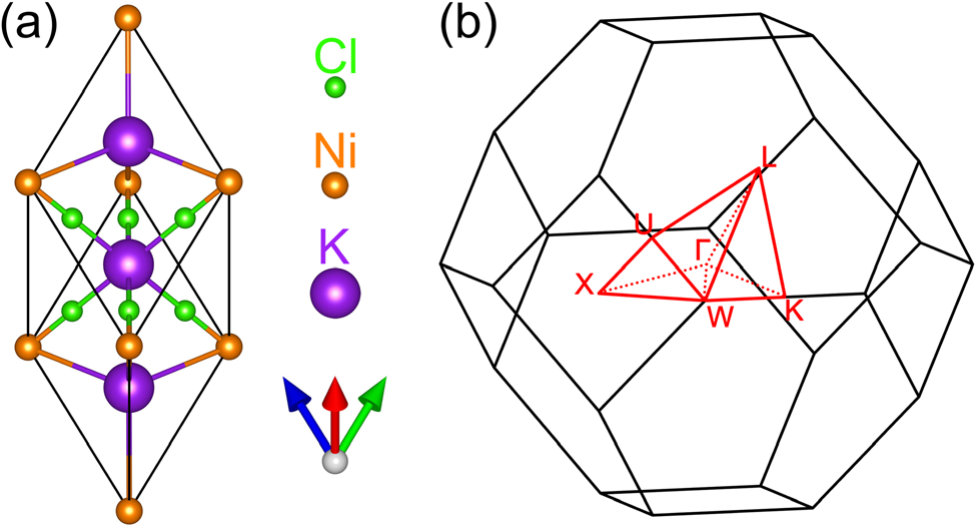
The primitive cell (a) and the corresponding Brillouin zone (b) for the ternary ferromagnetic compound K_3_NiCl_6_. The high symmetry paths and points are highlighted in red for clarity.

The ternary compound K_3_NiCl_6_ exhibits a cubic crystal structure characterized by the space group *Fm*3̄*m* (no. 225). Illustrated in Fig. 1a, the primitive lattice configuration features six Cl atoms positioned at the 24e Wyckoff site with coordinates (0.21906, 0, 0), a Ni atom situated at the 4a Wyckoff site (0, 0, 0), and two K atoms occupying the 4b (0.5, 0.5, 0.5) and 8c (0.25, 0.25, 0.25) Wyckoff sites, respectively. In this geometric arrangement, the Ni atom at the 4a site and the K atom at the 4b site are located at the centers of ideal octahedra, each being coordinated by six Cl atoms. Whereas, the K atom at the 8c site is positioned at the center of a tetrahedron, surrounded by four Ni atoms. For enhanced visualization of these atomic bond connections, several views of this primitive structure are presented in Fig. S1 of the ESI.† Given the inherent magnetic properties of Ni atoms, a thorough investigation into the magnetic configurations of K_3_NiCl_6_ is conducted. Four distinctive magnetic configurations are explored within a 2 × 2 × 1 supercell framework, encompassing a nonmagnetic state, a ferromagnetic state, and two antiferromagnetic states. These magnetic configurations are delineated in Fig. S2 of the ESI.† Assessment of the total energies associated with each configuration, see Table S1 of the ESI,† reveals that the ferromagnetic state stands out with the lowest energy, signifying its representation as the compound's magnetic ground state. The detailed magnetic moments and exchange coupling parameters under this ferromagnetic ground state are reported in Table S1 of the ESI.† Furthermore, the fully optimized structure yields an equilibrium lattice parameter of *a* = *b* = *c* = 7.451 Å for the primitive cell and *a* = *b* = *c* = 10.537 Å for the conventional cell.

Since K_3_NiCl_6_ has not yet been synthesized experimentally, assessing its stability is crucial. We evaluated its stability from both mechanical and dynamic perspectives, with comprehensive results available in the ESI.† The phonon dispersion spectrum, as derived by the finite-displacement method within density functional perturbation theory ^76^ and reported in Fig. S3 of the ESI,† exhibiting no soft modes, confirms its dynamic stability. Based on the stress–strain method, ^77^ the mechanical properties, including three independent elastic constants C_11_, C_12_, and C_44_, and, several other important mechanical parameters, like Young's modulus (*E*), shear modulus (*G*), bulk modulus (*B*), and Poisson's ratio (*ν*), have been obtained. Their values were are detailed in the Table S3 of the ESI.† They fulfill the elastic stability criteria: ^78,79^ C_11_ − C_12_ > 0, C_11_ + 2C_12_ > 0 and C_44_ > 0, indicating its mechanical stability. Together, these findings underscore the strong potential of K_3_NiCl_6_ for future experimental synthesis, supported by its robust mechanical and dynamic stability.

### Electronic structure and spin nodal box state

**Fig. 2:**
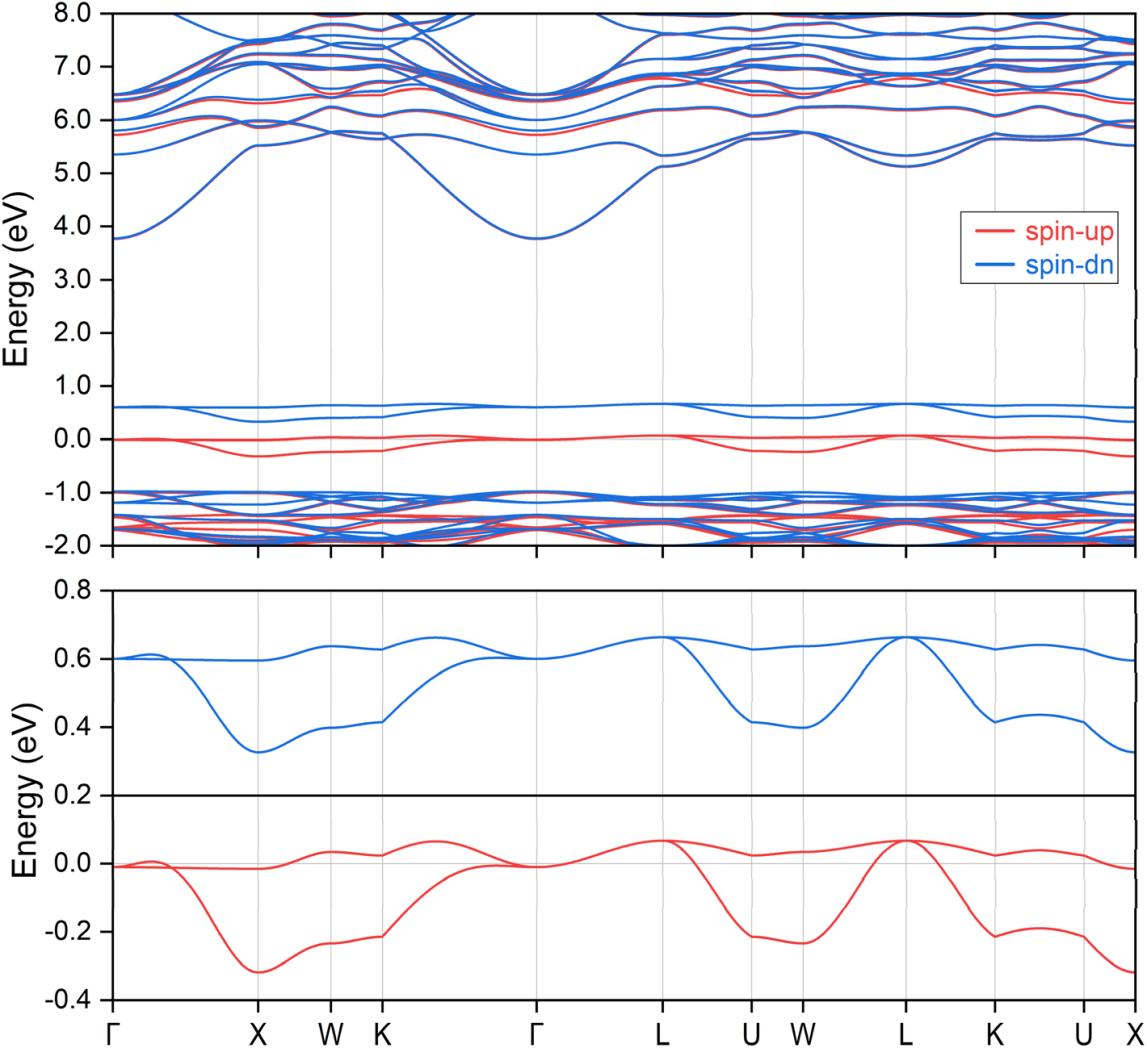
The spin polarized electronic band structures for the ternary ferromagnetic compound K_3_NiCl_6_. The Fermi energy level is shifted to 0 eV, as indicated by the horizontal line. Two local band structures in proximity to the Fermi energy level are further magnified at the bottom panel, in which multiple band crossings can be clearly observed.

We begin by presenting the calculated electronic band structure of the ternary compound K_3_NiCl_6_, as depicted in the top panel of Fig. 2, where the Fermi energy level is set to 0 eV. The selection of high-symmetry *k*-paths is based on the crystallographic data, as implemented in the Seek-Path code. ^80^ These k-paths are illustrated in Fig. 1b. The results reveal that K_3_NiCl_6_ exhibits a spin-polarized band structure, characterized by significant spin splitting in the two spin channels near the Fermi energy. Notably, four bands are observed in close proximity to the Fermi level, distinctly separated from other non-critical bands above and below them with large gaps. To enhance visualization, the local band structures of these four bands are further magnified in the lower panel of Fig. 2. Among these, two bands correspond to the spin-up channel, while the other two belong to the spin-down channel. These bands demonstrate multiple band crossing behavior and, more interestingly, both spin channels exhibit identical crossing conditions. For instance, in the spin-up channel, a linear band crossing is evident along the Γ-K path, while a doubly degenerate line is observed along the Γ-L path.

**Fig. 3:**
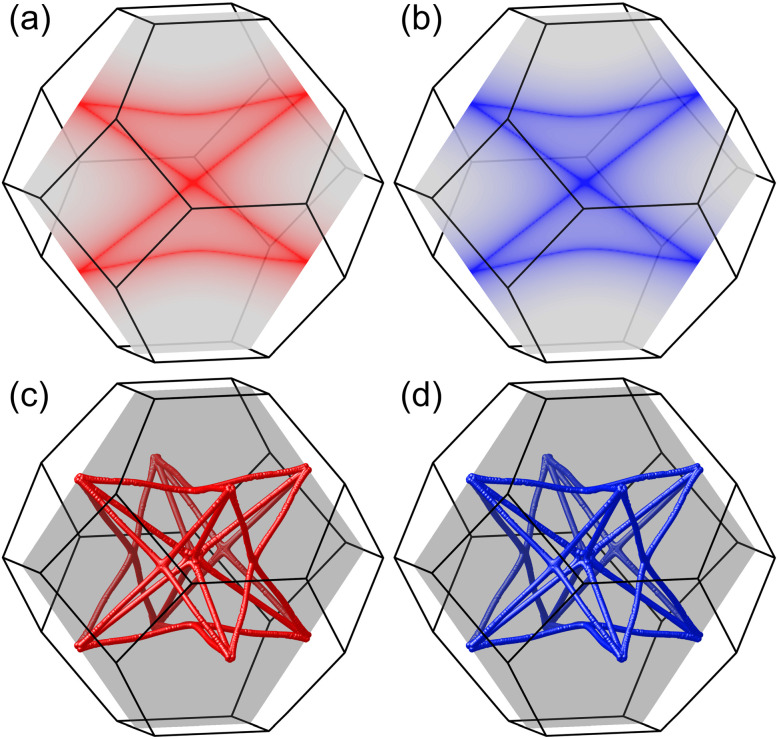
The distribution of the closed nodal line shape on a single (110) plane within the bulk Brillouin zone for both the spin-up (a) and spin-down (b) channels. The darkest color contour line signifies the zero energy difference between the two bands, as determined by precise DFT calculations. The spin nodal box structures formed by the amalgamation of all nodal lines across all six (110) planes for the spin-up (c) and spin-down (d) channels. The translucent gray area between the top and bottom panels is solely included for enhanced visual comparison.

Given that the same band configurations and crossings are observed for both bands within each spin channel, we can effectively treat each spin channel as a spinless system and subsequently analyze the corresponding topological configurations of the states for each spin. The doubly degenerate band observed along the Γ-L path corresponds to a nodal line, which is protected by the point group symmetry *C*_3v_. In contrast, the linear band crossing along the Γ-K path corresponds to a nodal point, protected by the point group symmetry *C*_2v_. Notably, since the same two bands are involved in these critical band crossing conditions, the resulting topological nodal line and nodal point are not isolated phenomena. A detailed examination of the two bands within the planes defined by the Γ-K and Γ-L paths reveals the formation of a closed nodal line shape, which resides on the (110) surface, determined by the mirror symmetry denoted as M_110_. This is illustrated in Fig. 3a and b for the spin-up and spin-down directions, respectively. Considering the *O*_h_ symmetry group of the cubic K_3_NiCl_6_ compound, we note that there are six symmetrically equivalent mirror planes associated with the M_110_ operations. As a result, all nodal lines present on the (110) surface converge and merge to form a nodal box, as depicted in Fig. 3c for the spin-up channel and Fig. 3d for the spin-down channel. Note that there is a very small difference between the nodal box distributions between the two spin channels, as illustrated in Fig. 4 of the ESI.† To the best of our knowledge, the spin nodal box structure identified in the current study is exceptionally rare, particularly in its manifestation across both spin channels. This complex nodal box configuration stands out when compared to other topological nodal line structures, as it offers significant advantages for both experimental characterization and practical applications. One notable benefit is that it does not necessitate a specific surface projection, thereby enhancing the versatility of its potential uses. It is important to mention that the effects of spin–orbit coupling (SOC) are not accounted for in the above analysis; a discussion of these effects will be presented in subsequent sections.

**Fig. 4:**
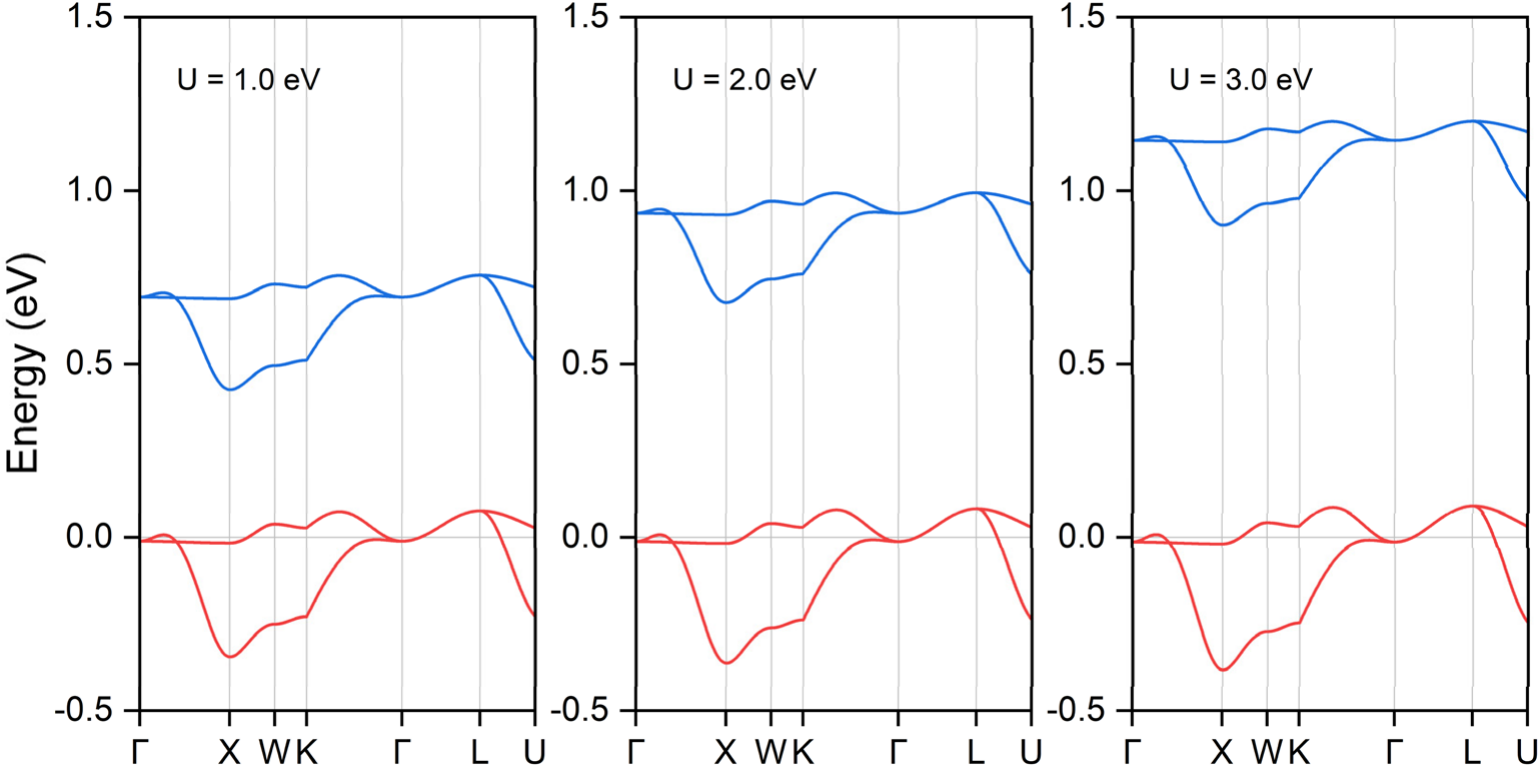
The local band structure in both spin channels along the Γ-X-W-K-Γ-L-U path for ternary ferromagnetic compound K_3_NiCl_6_ under DFT + *U* method. The Fermi energy level is shifted to 0 eV, as indicated by the horizontal line. Hubbard *U* values ranging from 1 to 3 eV were employed to accommodate strong Coulomb interactions. Only the bands related to the spin nodal box are shown.

### Effect of Hubbard *U* and spin–orbit coupling

To address the substantial Coulomb interaction and nonlocal correlation effects present in the transition metal Ni, we utilized the DFT + *U* method to further investigate the conditions for topological band crossings and the configuration of nodal complexes. A range of *U* values was explored, and their influence on the electronic band structure is illustrated in Fig. 4. For a clearer focus, only the bands along the X-Γ-L-K path are shown. Our findings indicate that variations in the *U* value do not affect the band crossing conditions in either spin channel, as the symmetry configurations remain unchanged. The spin nodal box structure persists in both spin channels; however, their energy distributions exhibit different behaviors. In the spin-up channel, the energy of the two bands remains relatively stable across different *U* values. In contrast, in the spin-down channel, an increase in the *U* value results in a slight upward shift of the nodal box state to higher energy levels. To ensure comprehensive analysis, we examined a broader range of *U* values, extending up to 6 eV, with the results presented in Fig. S5 of the ESI.† The band crossing conditions remain intact across this range. The trend observed shows that while the spin-up channel remains largely unaffected, the spin-down channel experiences further elevation in energy levels. This analysis underscores the differential impact of varying *U* values on the electronic structure of the material.

**Fig. 5:**
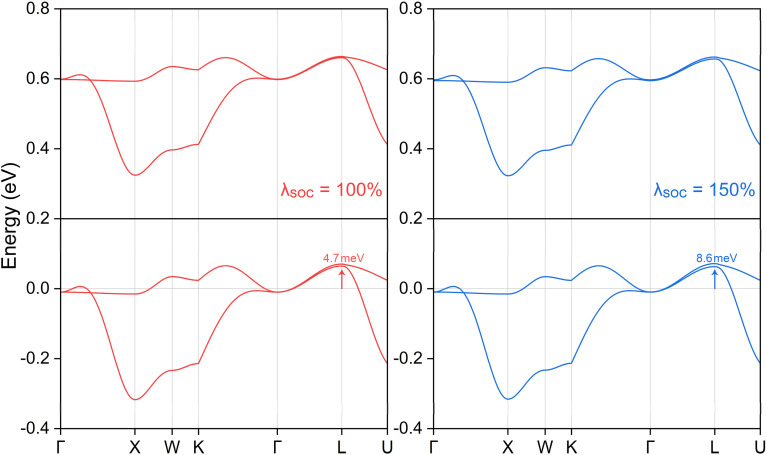
The calculated local band structure along the Γ-X-W-K-Γ-L-U path for ternary ferromagnetic compound K_3_NiCl_6_ under the influence of spin–orbit coupling effect. The Fermi energy level is shifted to 0 eV, as indicated by the horizontal line. Furthermore, the coupling intensity has been artificially increased to investigate extreme scenarios. The maximum opened band gap is situated at the L point, with the corresponding gap value explicitly indicated.

Additionally, it is crucial to consider the impact of spin–orbit coupling (SOC), which was not included in the initial analysis. Given the presence of heavy elements in the K_3_NiCl_6_ compound, SOC is anticipated to have a significant influence. Generally, SOC can perturb electronic systems by introducing an energy gap at critical topological states or by modifying fundamental topological features, potentially disrupting the original topological phase. We examined various magnetization directions, including [001], [010], [100], [110], [101], [011], and [111] axes. The calculated total energy values under different magnetization directions are reported in Table S4 of the ESI† and it can be found that the magnetization along the [100] direction corresponds to the ground state with the lowest energy. Under this [100] spontaneous magnetization, the electronic band structure near the Fermi level is depicted in Fig. 5. Note only the four bands involved for the topological structure are shown. Our findings indicate that the SOC effect has a negligible impact on the band structure, with any induced energy gap being less than 5 meV—insufficient for practical observation. To provide a thorough analysis, we also investigated two additional magnetization directions, [001] and [010]. The corresponding results, illustrated in Fig. S6 of the ESI,† confirm that the SOC effect remains negligible in these directions as well, with band gaps consistent with the [100] direction.

By applying both the Hubbard *U* correction and the spin–orbit coupling (SOC) effect, we determined the ground magnetization directions and total magnetic moments for different *U* values, see Tables S4 and S5 in the ESI.† Our findings show that the ground magnetization is not consistently along the [100] direction, though the energy differences between various directions are minimal. This allows for easy manipulation of magnetization using an external magnetic field, which is beneficial for experimental and technological applications requiring precise magnetic control. ^57^ Additionally, even when the SOC intensity is artificially increased beyond typical values, the maximum energy gap remains below 10 meV, see the right panel of Fig. 5. This enhanced intensity serves only as a hypothetical scenario to guide potential extreme cases. Consequently, the nodal complex structure retains its integrity, with its topological properties remaining largely unaffected by SOC. This preservation underscores the robustness of the nodal features in the presence of SOC, highlighting their potential for applications where topological stability is crucial. This finding contrasts with previously reported studies, ^81–84^ largely because the two bands responsible for the spin nodal box structure are primarily derived from the p orbitals of the lighter Cl atoms rather than the heavier Ni atoms. This stability of the spin nodal box against both Coulomb interactions and SOC underscores the resilience of K_3_NiCl_6_'s unique topological features. This robustness suggests a promising potential for experimental verification and further exploration of its novel properties in future research.

### Topological surface state

**Fig. 6:**
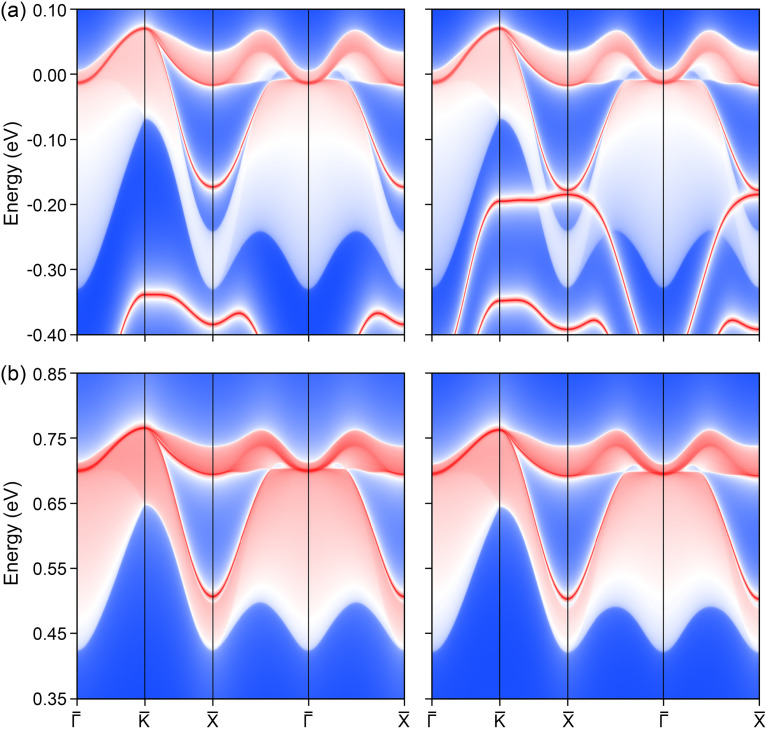
The (001) surface band spectrum for the ternary ferromagnetic compound K_3_NiCl_6_ in both the spin-up (a) and spin-down (b) channels. The right panel demonstrates the incorporation of spin–orbit coupling. Remarkable surface states emerge from diverse band crossings occurring along the entire Γ̄-K̄-X̄-Γ̄-X̄ path.

Topological materials are characterized by the presence of nontrivial surface states, which are inherently protected by their underlying topological order. In the case of the K_3_NiCl_6_ compound, the emergence of spin nodal complex structures strongly indicates the potential existence of drumhead surface states. To investigate this potential, we constructed a localized Wannier tight-binding Hamiltonian, emphasizing the contributions from the primary p orbitals of the Cl atoms for the two bands within the nodal box. The orbital-projected band structures, which provide detailed insight into these contributions, are presented in Fig. S7 and S8 of the ESI.† Using this Hamiltonian, we modeled a surface slab system and calculated the corresponding surface spectrum of K_3_NiCl_6_ along the (001) surface. The results, depicted in Fig. 6, reveal the surface states associated with the two spin nodal box structures. Specifically, Fig. 6a displays the surface states for the spin-up channel along the path Γ̄-K̄-X̄-Γ̄-X̄, both with and without the inclusion of SOC effects. Fig. 6b illustrates the corresponding surface states for the spin-down channel. Regarding to the surface Brillouin and the corresponding distribution of the high symmetry points and paths, please refer to Fig. S9 of the ESI.†

These calculations clearly demonstrate the presence of drumhead surface states that originate from and connect the band crossings within each spin nodal box. A noteworthy observation is the distinction in the surface states between the spin channels; the surface states in the spin-up channel are more separated from the bulk band projection compared to those in the spin-down channel. One more thing should be mentioned is the presence of extra surface states in the spin-up channel. With extended energy scale, we can see that these extra surface bands are not originated from the current spin nodal box, as demonstrated in Fig. S10 of the ESI.† Rather, they likely derive from trivial obstructed boundary states, characterized by their floating distribution without association to any specific band crossings. Considering the possible magnetization variation under different Hubbard *U* correction, the topological surface states along other two magnetization directions [010] and [001] have also been computed and obtained results, as reported in Fig. S11 and S12 of the ESI,† exhibit the same conditions as the [100] direction. More importantly, all the nontrivial surface states remain robust even in the presence of SOC, highlighting their stability and resilience. Furthermore, the extensive distribution of surface states across the Brillouin zone suggests a high electron density, which enhances the likelihood of their experimental detection. Consequently, these topological surface states hold significant promise for future technological applications, offering new avenues for exploration in the field of quantum materials.

Compared to previous studies on topological nodal line materials, the spin nodal box structures presented here offer several unique and advantageous features. One of the most notable distinctions is that the nodal box configuration in K_3_NiCl_6_ consists of only two bands, which corresponds to the simplest band crossing situation. Additionally, the nodal box with spin polarized behavior can provide addition spin degree for the manipulation of the topological properties. More importantly, all the spin box structures are accompanied by prominent surface states that not only remain separated from the bulk band projection but also demonstrate remarkable robustness against SOC effects. Besides, the broad distribution of surface states across the Brillouin zone enhances their potential for experimental detection and practical application, particularly in environments of heterojunction scheme. The inherent properties of K_3_NiCl_6_ extend the range of observable topological behaviors, positioning it as a versatile candidate for advanced applications where directional dependency might otherwise limit performance. The robustness of these surface states, coupled with their distinct spin polarization and broad distribution, underscores the material's potential in engineering environments that demand high precision and stability. These characteristics suggest that K_3_NiCl_6_ could be instrumental in the development of spin-related topological properties, where directional control and topological protection are essential.

## Conclusions

In this study, we present a comprehensive theoretical investigation of the topological properties of the ternary compound K_3_NiCl_6_. Under the ferromagnetic ground state, we identify two distinct bands situated near the Fermi energy level within each spin channel. These bands are well-separated from the trivial bands both above and below, indicating a significant topological distinction. The interaction between these two bands gives rise to multiple band crossings, which collectively form an exotic spin nodal box structure. This structure is protected by the equivalent six mirror operation, denoted as M_110_, inherent to the cubic *O*_h_ symmetry group. Notably, when incorporating the Hubbard *U* correction, the band crossing characteristics in both spin directions remain intact even under the variation of *U* values, thereby preserving the essential features of the topological structure. To further elucidate the topological surface spectrum, we construct a localized Wannier tight-binding Hamiltonian. This approach reveals that the spin nodal complex structure is characterized by prominent surface states exhibiting a broad spatial distribution, which not only enhances the feasibility of experimental observation but also opens avenues for potential applications. In contrast to previously studied nodal line configurations, the spin nodal complex in K_3_NiCl_6_ exhibits multiple surface states, thereby augmenting its versatility and functional efficacy. Moreover, the topological robustness of this configuration is maintained even in the presence of spin–orbit coupling effects. The stability of these surface states under SOC is fundamentally attributed to the contributions from the light element orbitals associated with the relevant topological bands. In summary, the K_3_NiCl_6_ compound demonstrates a diverse array of advantages and robust topological properties, positioning it as a promising candidate for experimental investigation. Its unique spin nodal box structure, combined with the stability of surface states in the presence of SOC effects, highlights its potential for immediate experimental scrutiny and further exploration, particularly in relation to its magnetic properties.

## Data availability

The data that support the findings of this study are available from the corresponding author upon reasonable request.

## Author contributions

Yang Li: conceptualization, methodology, software, visualization, investigation, writing – original draft preparation, validation, writing – reviewing and editing.

## Conflicts of interest

The authors declare that they have no known competing financial interests or personal relationships that could have appeared to influence the work reported in this paper.

## Supplementary Material

RA-014-D4RA06808D-s001
