# Supplementary material for: Dual spin nodal box structure in ternary ferromagnet K3NiCl6 with broad topological surface states
Source: RSC Adv. 2024 Nov 15;14(49):36497–506. doi: 10.1039/d4ra06808d (PMC11565218; doi:10.1039/d4ra06808d)
Supplement: RA-014-D4RA06808D-s001 [file RA-014-D4RA06808D-s001.pdf]

Supplementary Information (SI) for RSC Advances.
This journal is © The Royal Society of Chemistry 2024

Dual spin nodal box structure in ternary ferromagnet K_3NiCl_6 with broad topological surface states

Yang Li[1,2,*]

[1] Aviation and Automobile School, Chongqing Youth Vocational & Technical College, Chongqing, China.

[2] College of Physics, Chongqing University, Chongqing, China.

[*] Emails: liyang@cqwu.edu.cn

Supplementary materials

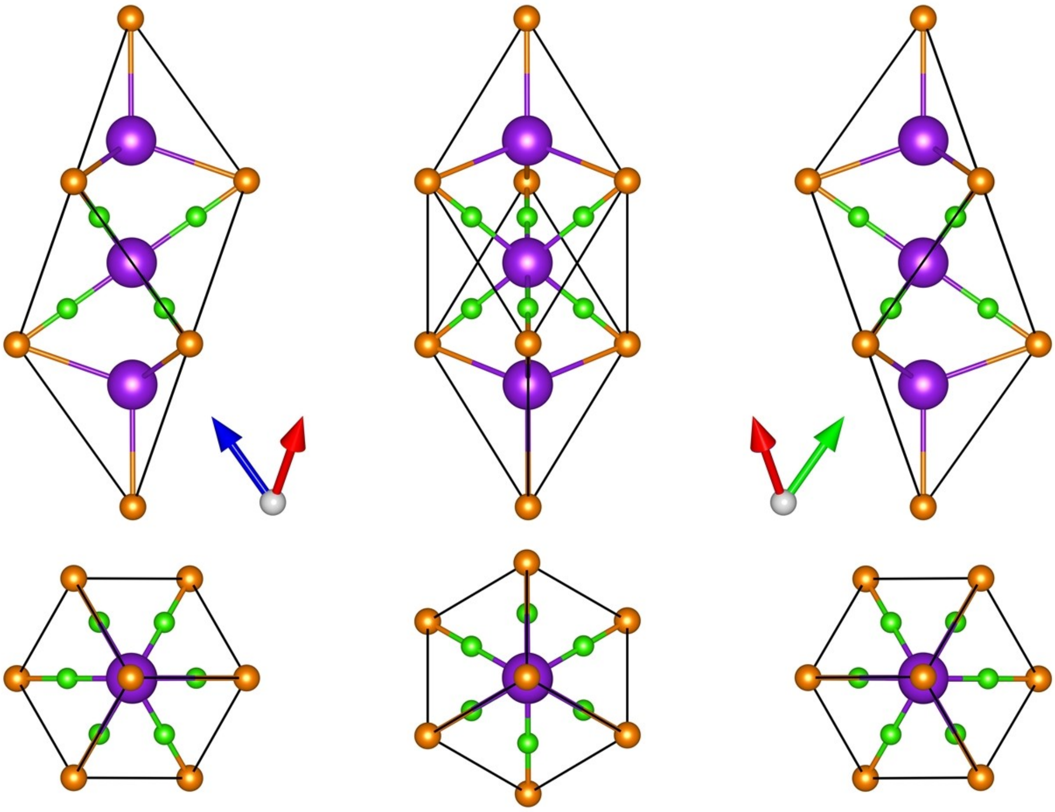

Figure S1: The primitive cell for the ternary ferromagnetic compound K_3NiCl_6 under different view directions.

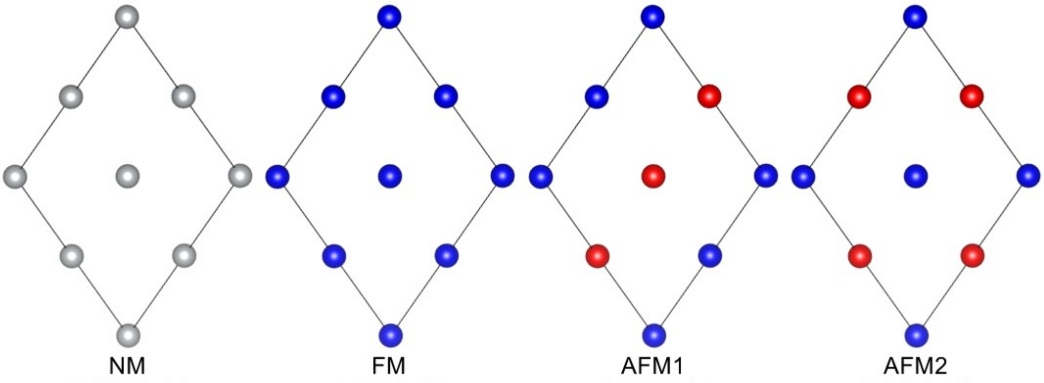

Figure S2: The determination of the magnetic ground state. Four magnetic configurations have been considered within the 2×2×1 super cell for the ternary compound K_3NiCl_6, including a nonmagnetic state (NM), a ferromagnetic state (FM), and two antiferromagnetic states (AFM1 and AFM2).

Table S1: The calculated total energy for the ternary compound K_3NiCl_6 under different magnetic configurations and Hubbard U values, including a nonmagnetic state (NM), a ferromagnetic state (FM), and two antiferromagnetic states (AFM1 and AFM2). All units are in eV.

U	NM	FM	AFM1	AFM2
0	-129.92650387	-130.37598263	-130.33907432	-130.36583783
1	-127.53794896	-128.25866331	-128.20413630	-128.23976407
2	-125.18643464	-126.25504395	-126.18571625	-126.23018987
3	-122.87266109	-124.39084332	-124.30830860	-124.36164512
4	-120.59756899	-122.69169927	-122.59702055	-122.65917156
5	-118.36243350	-121.17672655	-121.07046360	-121.14217675
6	-116.16845808	-119.85908214	-119.82164481	-119.73963995

Table S2: The calculated total and local magnetic moments and coupling parameters for the ternary compound K_3NiCl_6 under different Hubbard U values.

U	M_{total}	M_K	M_{Ni}	M_{Cl}	J_1	J_2
eV	μ_B				meV	
0	0.9994	0.0000	0.7060	0.0333	0.59518	2.58896
1	0.9996	0.0000	0.8000	0.0590	0.90311	2.71722
2	1.0000	0.0000	0.9120	0.0480	0.92082	2.59736
3	0.9995	0.0000	1.0390	0.0440	0.83576	2.37550
4	1.0000	0.0000	1.1730	0.0630	0.73327	2.14900
5	0.9998	0.0000	1.3050	0.0810	0.63122	1.97976
6	0.9908	0.0000	1.4340	0.0990	0.56502	1.86005

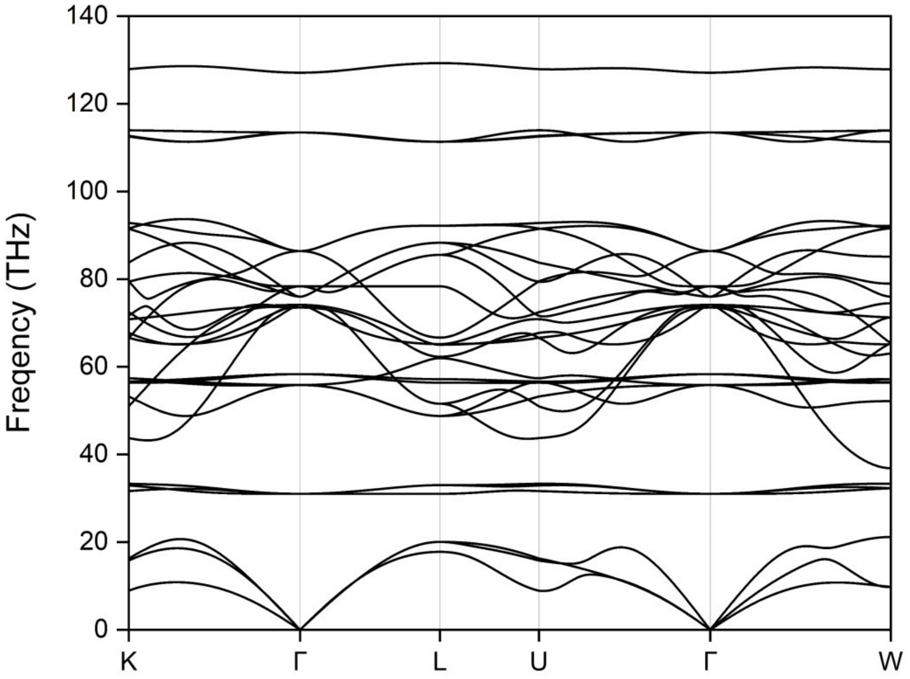

Figure S3: The calculated phonon spectrum for the ternary compound K_3NiCl_6.

Table S3: The calculated various elastic constants (C_{11}, C_{12}, and C_{44}), Young's modulus (E), shear modulus (G), and Poisson's ratio (ν) for the ternary compound K_3NiCl_6.

C_{11}	C_{12}	C_{44}	E	G	B	ν
(GPa)						
41.299	17.739	9.616	27.548	10.430	25.593	0.321

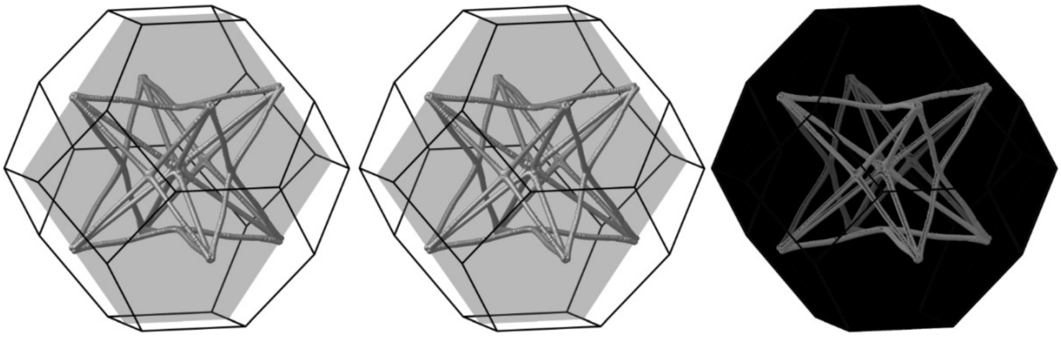

Figure S4: The nodal box distributions in the two spin channels and their difference in the Brillouin zone. The gray scale is applied to provide the same color reference for better comparison. The slight white color in the right panel with most of black color indicates the difference between the two spin channels.

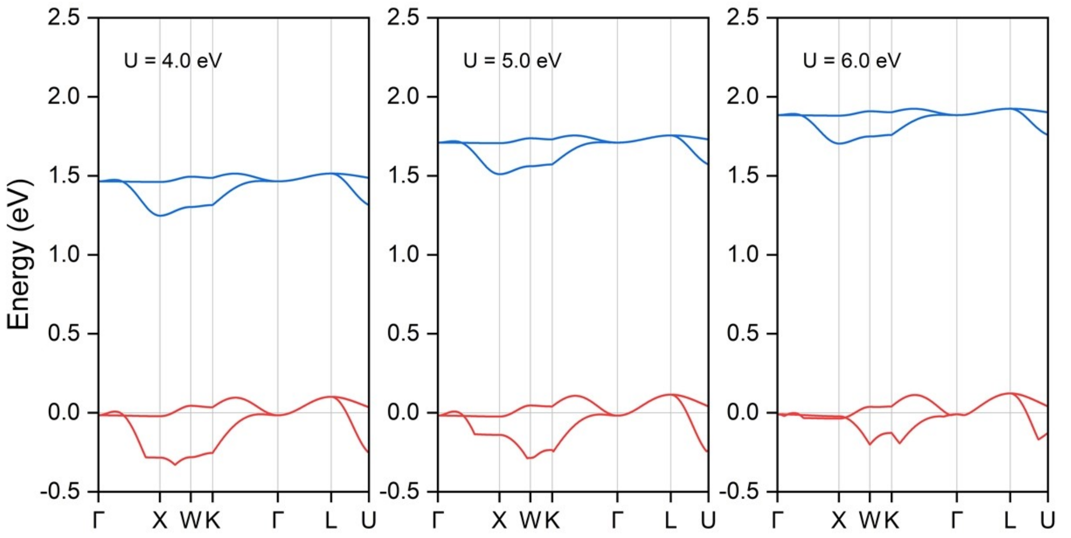

Figure S5: The local band structure in both spin channels along the Γ-X-W-K-Γ-L-U path for ternary ferromagnetic compound K_3NiCl_6 under DFT+U method. The Fermi energy level is shifted to 0 eV, as indicated by the horizontal line. Hubbard U values ranging from 4 to 6 eV were employed to accommodate strong Coulomb interactions. Only the bands related to the spin nodal box are shown.

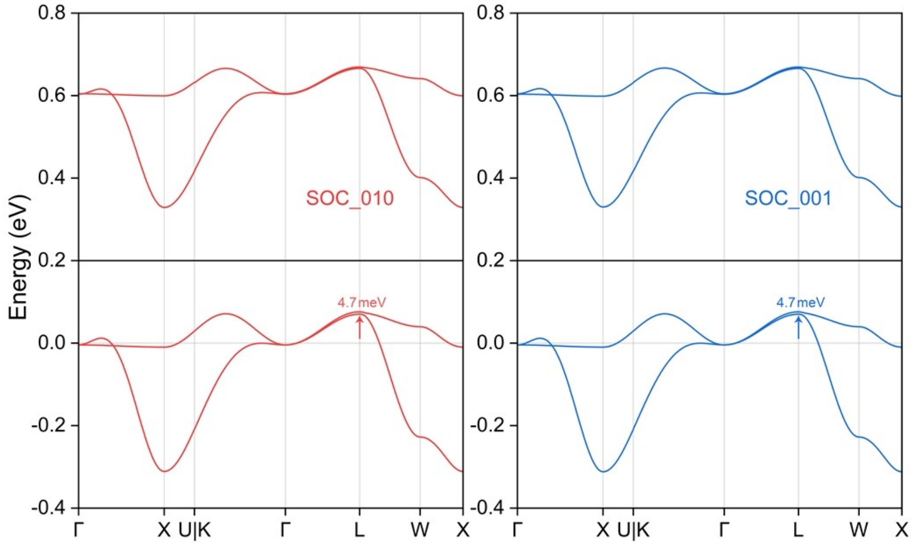

Figure S6: The calculated local band structure along the Γ-X-W-K-Γ-L-U path for ternary ferromagnetic compound K_3NiCl_6 under [010] and [001] magnetization directions. The Fermi energy level is shifted to 0 eV, as indicated by the horizontal line. The maximum opened band gap is situated at the L point, with the corresponding gap value explicitly indicated.

Table S4: The calculated total energy (eV) under different magnetization directions with different Hubbard U corrections. The red color indicates the ground energy state.

U	E_{001}	E_{010}	E_{100}	E_{110}	E_{101}	E_{011}	E_{111}
0 eV	-32.62037829	-32.62037791	-32.62037873	-32.62037806	-32.62037815	-32.62037780	-32.62037709
U	E_{001}	E_{010}	E_{100}	E_{110}	E_{101}	E_{011}	E_{111}
1 eV	-32.08976873	-32.08976834	-32.08976835	-32.08976819	-32.08976848	-32.08976838	-32.08976668
U	E_{001}	E_{010}	E_{100}	E_{110}	E_{101}	E_{011}	E_{111}
2 eV	-31.58731477	-31.58731472	-31.58731519	-31.58731576	-31.58731582	-31.58731589	-31.58731229
U	E_{001}	E_{010}	E_{100}	E_{110}	E_{101}	E_{011}	E_{111}
3 eV	-31.11977134	-31.11977149	-31.11977218	-31.11977194	-31.11977192	-31.11977176	-31.11976735
U	E_{001}	E_{010}	E_{100}	E_{110}	E_{101}	E_{011}	E_{111}
4 eV	-30.69358290	-30.69358308	-30.69358617	-30.69358058	-30.69358145	-30.69358147	-30.69357441
U	E_{001}	E_{010}	E_{100}	E_{110}	E_{101}	E_{011}	E_{111}
5 eV	-30.31365378	-30.31365164	-30.31365459	-30.31364610	-30.31364599	-30.31364619	-30.31364104
U	E_{001}	E_{010}	E_{100}	E_{110}	E_{101}	E_{011}	E_{111}
6 eV	-29.98338469	-29.98338037	-29.98338447	-29.98338381	-29.98338186	-29.98338109	-29.98337895

Table S5: The calculated total magnetic moment (μ_B) under different magnetization directions with different Hubbard U corrections.

U	M_{001}	M_{010}	M_{100}	M_{110}	M_{101}	M_{011}	M_{111}
0 eV	1.0011	1.0011	1.0011	0.7079	0.7079	0.7079	0.5780
U	M_{001}	M_{010}	M_{100}	M_{110}	M_{101}	M_{011}	M_{111}
1 eV	1.0004	1.0004	1.0004	0.7074	0.7074	0.7074	0.5776
U	M_{001}	M_{010}	M_{100}	M_{110}	M_{101}	M_{011}	M_{111}
2 eV	0.9997	0.9997	0.9997	0.7069	0.7069	0.7069	0.5772
U	M_{001}	M_{010}	M_{100}	M_{110}	M_{101}	M_{011}	M_{111}
3 eV	0.9994	0.9994	0.9994	0.7067	0.7067	0.7067	0.5770
U	M_{001}	M_{010}	M_{100}	M_{110}	M_{101}	M_{011}	M_{111}
4 eV	1.0002	1.0002	1.0002	0.7072	0.7072	0.7072	0.5774
U	M_{001}	M_{010}	M_{100}	M_{110}	M_{101}	M_{011}	M_{111}
5 eV	1.0037	1.0035	1.0037	0.7096	0.7096	0.7096	0.5795
U	M_{001}	M_{010}	M_{100}	M_{110}	M_{101}	M_{011}	M_{111}
6 eV	1.0110	1.0108	1.0110	0.7148	0.7147	0.7147	0.5835

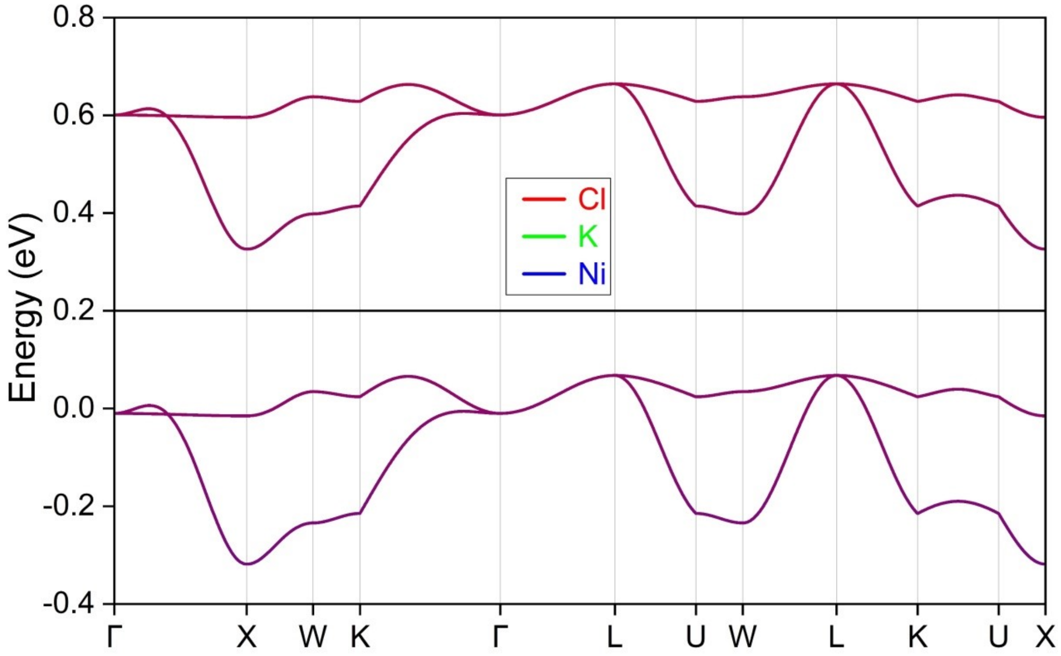

Figure S7: The projection for the two bands related to the spin nodal box structure in both spin channels. The line color is weighted with the different element contributions.

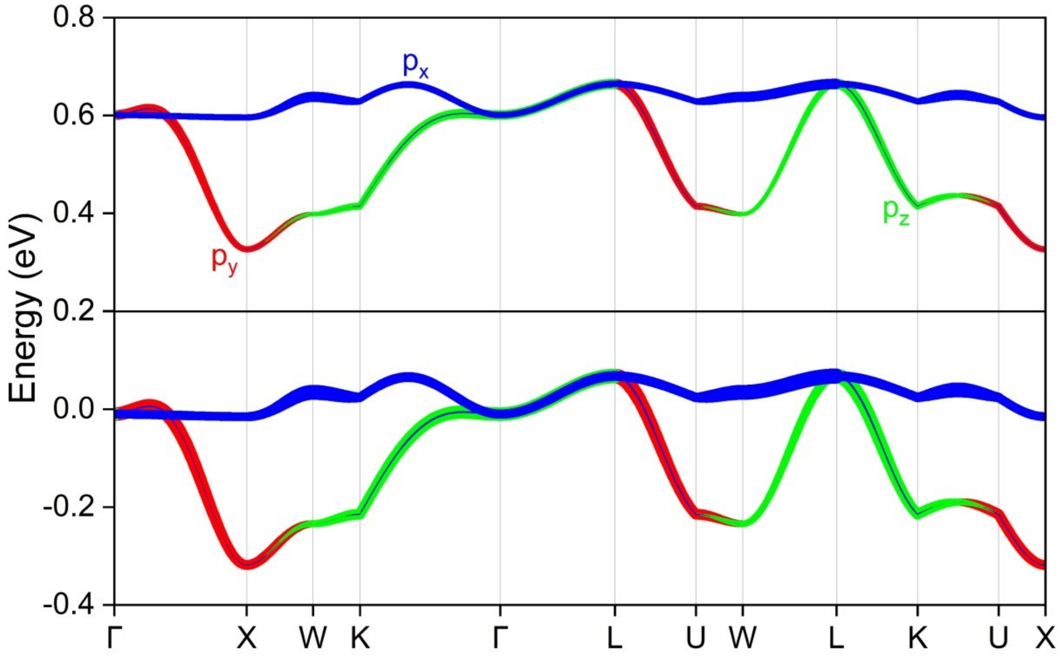

Figure S8: The orbital projection for the two bands related to the spin nodal box structure. Only the major contributions from the p orbital of Cl elements are selected and shown.

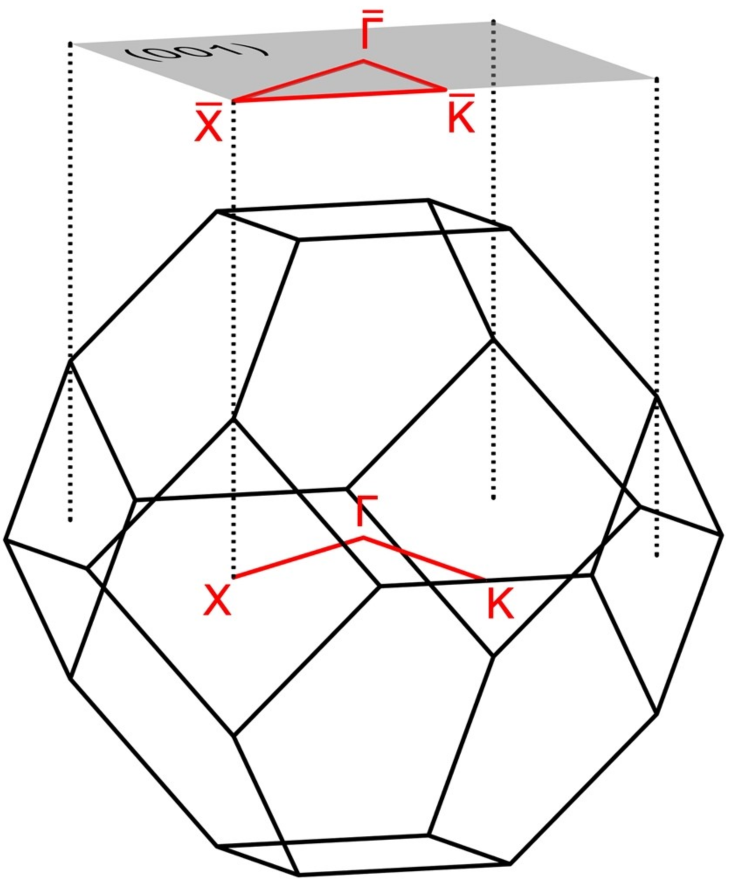

Figure S9: The (001) surface Brillouin zone and the high symmetry points and paths, with correspondence from the bulk Brillouin zone.

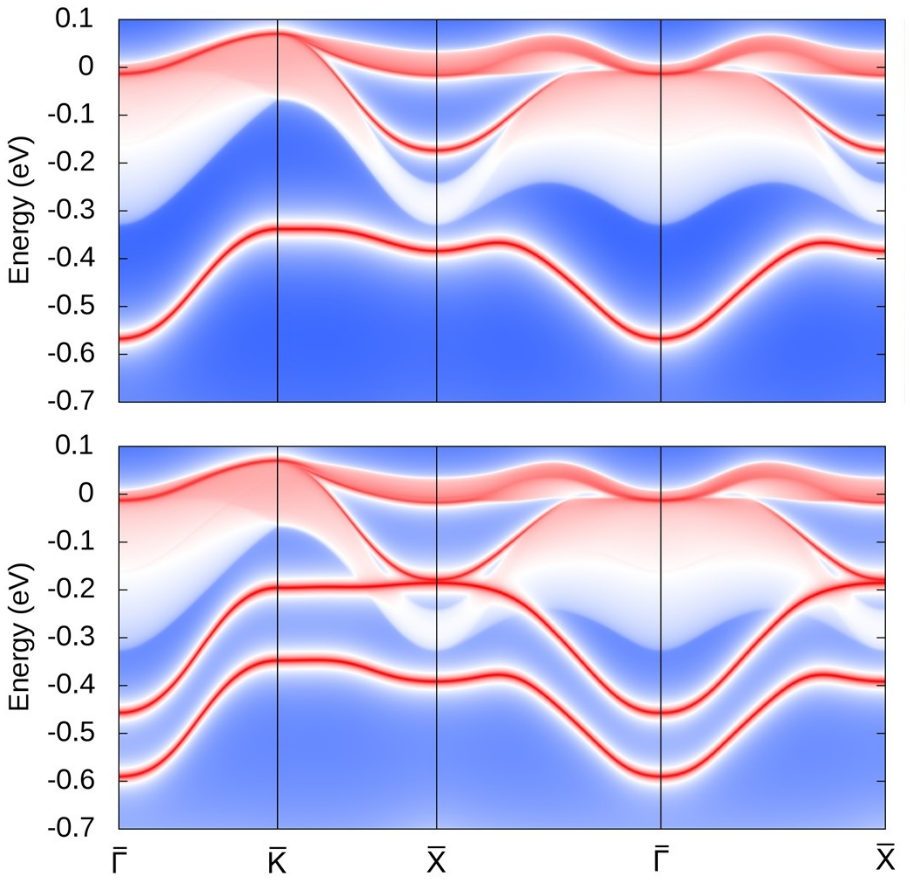

Figure S10: The (001) surface band spectrum for the ternary ferromagnetic compound K_3NiCl_6 in the spin-up channels with extended energy scale. The extra surface states with floating behavior are from the trivial obstructed boundary states.

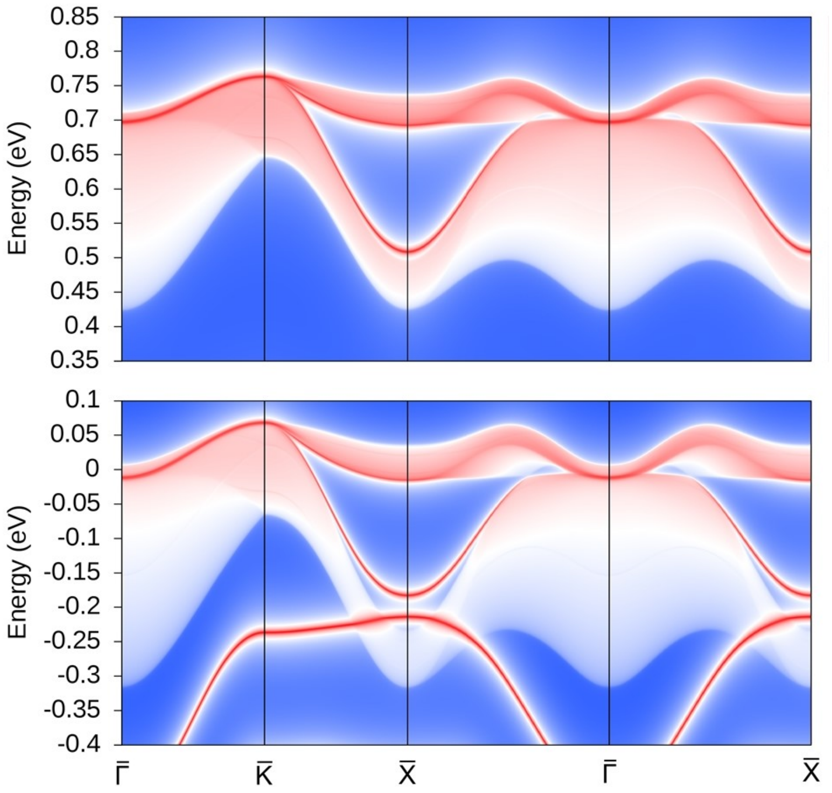

Figure S11: The (001) surface band spectrum for the ternary ferromagnetic compound K3NiCl6 under [010] magnetization direction.

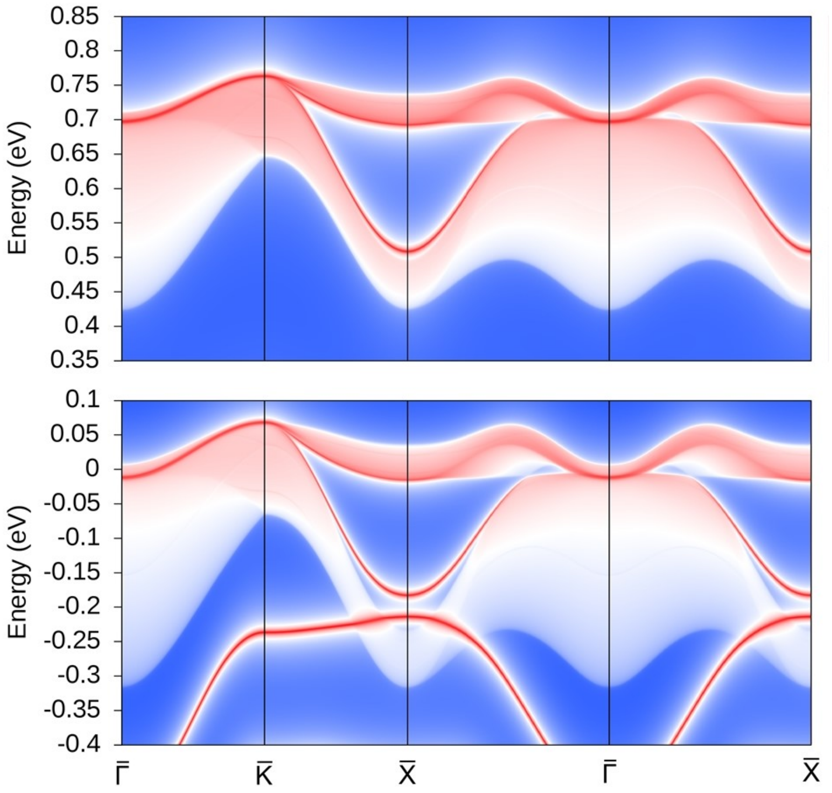

Figure S12: The (001) surface band spectrum for the ternary ferromagnetic compound K3NiCl6 under [001] magnetization direction.
